# The Physiological Effect of a Holoparasite Over a Cactus Along an Environmental Gradient

**DOI:** 10.3389/fpls.2021.763446

**Published:** 2021-11-18

**Authors:** Carmen Gloria Ossa, Daniela Aros-Mualin, María Isabel Mujica, Fernanda Pérez

**Affiliations:** ^1^Facultad de Ciencias, Instituto de Biología, Universidad de Valparaíso, Valparaíso, Chile; ^2^Centro de Investigación y Gestión de Recursos Naturales, Universidad de Valparaíso, Valparaíso, Chile; ^3^Department of Systematic and Evolutionary Botany, University of Zurich, Zurich, Switzerland; ^4^Instituto de Ecología y Biodiversidad, Santiago, Chile; ^5^Departamento de Ecología, Facultad de Ciencias Biológicas, Pontificia Universidad Católica de Chile, Santiago, Chile

**Keywords:** cactus, holoparasite, functional traits, isotope ecology, environmental gradient, CAM, plant-plant interaction, mistletoe

## Abstract

*Echinopsis chiloensis* is an endemic cactus from Chile, distributed in a temperature and rainfall gradient between 30° and 35° South latitude, with mean temperatures increasing and precipitation decreasing toward the north. It is the main host of the mistletoe *Tristerix aphyllus*, a holoparasite completely dependent on the cactus for water, carbon, and minerals. In this study, we investigated the consequences of parasitism over the fitness and physiology of this cactus throughout its distribution range and how it is affected by the environment. We measured five functional traits in eight populations latitudinally distributed, the first three only for the host: reproductive fitness, stomatal traits (density and size), and photosynthesis (during winter and summer); and the last two for the host and parasite: stable isotopes (∂^13^C and ∂^15^N), and nutrients (carbon and nitrogen content). The results showed a negative effect of parasitism over fitness of infected cacti. However, the higher nitrogen concentrations in cactus tissues toward the south improved overall fitness. Regarding photosynthesis, we only observed a negative effect of parasitism during the dry season (summer), which is also negatively affected by the increase in summer temperatures and decrease in winter rainfall toward the north. There were no differences in nutrient concentration or in the isotopic signature of healthy and infected cacti. Conversely, we observed a higher carbon and lower nitrogen concentration in mistletoes than in cacti regardless of latitude. The loss of temperature seasonality toward the north increases the C:N ratio, and the values between the parasite and its host diverge. ∂^15^N was similar between parasites and hosts while ∂^13^C of the parasite was enriched when compared to its host. Overall, the infection by *T. aphyllus* affects *Echinopsis chiloensis* fitness but showed no strong effects over the cactus physiology, except for the summer photosynthesis. Therefore, our data revealed that *E. chiloensis* response to *T. aphyllus* infection is sensitive to environmental changes in a way that could be strongly impacted by the desertification projected for this area due to climate change.

## Introduction

Mistletoes are aerial parasites with approximately 1,300 species distributed in three families: Misodendraceae, Loranthaceae, and Santalaceae (Glatzel and Geils, 2009; Nickrent, 2020). Most of the species are dispersed by birds and have seeds with viscin that allows them to adhere to the host. Once attached to it, the mistletoe penetrates the host’s tissue with a specialized structure called haustorium. The haustorium of hemiparasites invades the xylem tissue, absorbing mainly water and nutrients (Tìšitel et al., 2010; Bell and Adams, 2011; Tìšitel, 2016) and sometimes competing for light with their hosts (Tìšitel et al., 2010). In contrast, the holoparasites lack photosynthetic tissue and so invades the host’s xylem together with the phloem, absorbing not only water and nutrients, but also carbohydrates (Watling and Press, 2001; Tìšitel, 2016). This invasion of the vascular tissue could significantly impact their host’s form, growth, physiology, and reproduction (Press et al., 1999; Howell and Mathiasen, 2004).

The invasion of the host’s tissues by a holoparasite has been reported to produce varying effects on its photosynthetic rate (Jeschke et al., 1997; Fernandes et al., 1998; Hibberd et al., 1998). The magnitude of the reaction will depend on the nature of the interaction (Jeschke et al., 1997; Fernandes et al., 1998; Hibberd et al., 1998). For example, three holoparasitic species of *Cuscuta* have been known to cause negative effects on host’s photosynthesis. *Cuscuta australis* and *Cuscuta campestris* also have a negative effect on stomatal conductance and transpiration rates over its host *Mikania micrantha* (Shen et al., 2007, 2011; Le et al., 2015). It has been hypothesized that these differences result from the holoparasite acting as an additional sink, for which the host would sometimes compensate by increasing the photosynthetic rate, increasing the leaf area, or delaying foliar senescence (Casadesús and Munné-Bosch, 2021). This overcompensation could be more harmful to the host rather than to the parasite (Watling and Press, 2001), which would, in any case, reduce its growth and yield (Watling and Press, 2001; Zhuang et al., 2018).

The variation in environmental conditions can also influence the host’s response to parasitism, which will be subordinate to the availability of nutrients and water. It has been observed that nitrogen and water supplementation has mixed effects on the parasite-host relationship. For instance, water and nitrogen supplementation has been reported to reduce the effects of parasitism, both for holo- and hemiparasites, presumably prompted by an increase in host vigor (Jeschke and Hilpert, 1997; Shen et al., 2007, 2011, 2013). However, in some cases, as is for the hemiparasite of cereals *Striga hermonthica*, nutrient supplementation shows no improvement in the hosts condition (Aflakpui et al., 1998, 2002, 2005; Sinebo and Drennan, 2001). The same was observed in *Ulex europaeus* and *Acacia paradoxa* when infected with the hemiparasite *Cassytha pubescens* (Cirocco et al., 2017) and in hosts infected by the holoparasite *Rhinanthus alectorolophus*, which produced a negative effect regardless of supplementation of water or nitrogen (Korell et al., 2019). Solely regarding water stress, irrigation significantly reduces the harmful effect of the holoparasite *Cuscuta australis* over the photosynthesis of *Mikania micrantha* (Le et al., 2015). However, it decreases the biomass of *Verbesina alternifolia* when infected by *Cuscuta* (Evans and Borowicz, 2013, 2015). Environmental gradients also provide for attractive natural studies, although scarcely explored due to the difficulty of finding a host-parasite interaction along a gradient. Yang et al. (2012), for example, evaluated the parasite-host interaction through an elevational gradient going from the lowlands at the margins of the Gobi Desert to the highlands in the Tibetan Plateau. They compared the isotopic composition of the parasite with its host between sites where soil varies in salt content and compaction. Their findings show significant differences in the highlands between the host and parasite isotopic composition (∂^13^C and ∂^15^N), but not in the lowlands. This result confirms the influence of the environment while evidencing its interaction with the parasite’s effect over the host.

Although Cactaceae and parasitic plants are common in the Americas, *Tristerix aphyllus* is the only one capable of parasitizing Cactaceae (Mauseth et al., 2002), specifically columnar cacti of the genera *Echinopsis* and *Eulychnia* (Follman and Mahú, 1964; Mauseth, 1990). *T. aphyllus* is an endophytic holoparasite of the stem, and the only species of the genus that lost its leaves. This means that it depends completely on water, minerals, and photosynthates of the host. There are several challenges that *T. aphyllus* must overcome when parasitizing a cactus. For example, cactus have a minimal water and carbohydrate transportation rate, so *T. aphyllus* grows from a “reservoir” of water and nutrients of the host (Mauseth et al., 1984). Unlike other parasites, *T. aphyllus* invades the host primarily through the stomata because the thickness of the cactus cuticle makes it nearly impossible to break. Once inside its host, the endophyte of the parasite comprises a network of filaments that penetrates mainly through the intracellular spaces toward the host’s phloem and xylem, allowing for the host’s normal development (Mauseth et al., 2002). Seventeen months after the infection (Botto-Mahan et al., 2000), it emerges only the reproductive tissue of *T. aphyllus* from the cactus. The interaction between *T. aphyllus* and its principal host, *Echinopsis chiloensis*, is distributed along a marked precipitation and temperature gradient, providing an exceptional research opportunity. While the effect on fitness of *E. chiloensis* caused by *T. aphyllus* has been documented (Silva and Martínez del Río, 1996; Medel, 2000), little is known about the physiological consequences. Therefore, this work aims to examine the consequences of the parasite *T. aphyllus* over the physiological and isotopic performance of *E. chiloensis* in a gradient of precipitation and temperature associated with the host’s distribution range.

## Materials and Methods

### Study Area

**FIGURE 1:**
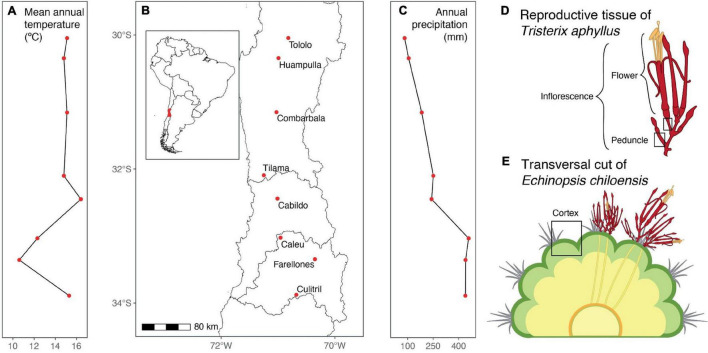
Spatial range studied of the interaction of the holoparasite *Tristerix aphyllus* with its host *Echinopsis chiloensis* and its general climatic conditions. **(A)** Mean annual temperature per population, **(B)** map of the analyzed populations, **(C)** annual precipitation per population, **(D)** diagram of *Tristerix aphyllus* inflorescence, and **(E)** diagram of a cross section of *Echinopsis chiloensis* infected by *T. aphyllus.*

We worked in eight populations of *Echinopsis chiloensis* spread all over the host-parasite interaction distribution (Figure 1B) and covering a precipitation and temperature gradient from north to central Chile (Figures 1A,C). All populations have a Mediterranean climate, characterized by cold and rainy winters and warm and arid summers. The annual precipitation throughout this gradient goes from 77 to 523 mm, and the mean annual temperatures from 11 to 16°C (Figure 1 and Supplementary Figure 6).

### Trait Measurement

We measured a set of traits associated with plant fitness and those that have a strong relationship with resource acquisition. Particularly ones that could serve as stress indicators when comparing non- parasitized (healthy) and parasitized (infected) cacti, such as the production of fruits, photosynthesis, stomatal traits, isotopes, and nutrients.

Fitness was measured as the number of fruits per individual plant (see Silva and Martínez del Río, 1996) in 15 healthy and 15 infected cacti by location with a comparable estimated age (around 5 m, and four or five branches). In the case of infected cacti, we selected individuals with only one exophytic parasite of similar size (between 30 and 40 cm of diameter). This allows us to standardize the parasite load and thus disentangle the effect of climate versus parasites over the cactus. It is also important to note that once a cactus is infected, it takes 17 months for the reproductive section of the parasite to emerge (Botto-Mahan et al., 2000). However, it is possible to observe a characteristic swelling on the arm of the cactus before emergence. Moreover, during the non-reproductive season of the parasite, it is possible to see branches from previous reproductive periods. For this reason, we avoided cacti presenting any anomaly or old reproductive branches.

For all measurements hereafter, we selected 20 individuals of *E. chiloensis* in each locality, 10 healthy and 10 infected cacti, following the same criteria as previously stated. CAM plants store CO_2_ as organic acids in vacuoles during the night; therefore, the photosynthetic activity can be indirectly determined by comparing the tissues’ acidity (mmol H+/gr) collected at sunset and dawn (Nobel, 1999; Lambers et al., 2008). To this end, samples from the cortex (Figure 1E) were collected at sunset and dawn and stored first in liquid nitrogen for transportation and later at −80°C in the laboratory. Next, pieces of 0.5–1 g of the frozen samples were homogenized with an Ultra Turrax Homogenizer (20,000 rpm) in 30 ml of distilled water. Lastly, the liquid was treated with NaOH 0.01 N until pH 7.0 was reached (potentiometer Hanna pH 21, keeping a steady temperature of 4°C). Unfortunately, the cold chain failed for the samples from Tololo (TO) and could not be included in this analysis.

One second set of cortex samples together with its corresponding parasite floral pedicel were stored in silica gel to assess stomatal traits, isotopic and nutrient analysis. The pedicel refers to the stem that attaches a single flower to the inflorescence. Regarding the stomatal traits, *Echinopsis* presents sunken stomata to minimize water loss, making it impossible to see them from the outside of the cuticle; hence samples had to be first treated with sodium hypochlorite 80% to dissociate the epidermis so we could see the stomata from the underside. Afterward, they were stained with methylene blue and mounted for observation using an optical microscope Olympus CX31 at 10x equipped with a digital camera. The photos were processed using Image J Software (Schneider et al., 2012). The same photos were used to: (a) quantify the stomatal density (SD = stomatal number/mm^2^), (b) guardian cells length (GL; μm), and (c) the stomatal conductance index (PCI = SD × GL × 2 × 10^–4^; Holland and Richardson, 2009).

Isotopic (∂^13^C and ∂^15^N) and nutrient analysis (%C and %N) were analyzed for healthy and infected cactus together with its respective parasitic plant. The material was analyzed in the Laboratory of Biogeochemistry and Applied Stable Isotopes, Pontificia Universidad Católica de Chile.

### Data Analysis

We explored the relationship of parasitism and climate over *E. chiloensis* to test which one was driving the physiological performance of the cactus. We used five climatic variables from Pliscoff et al. (2014), namely mean annual temperature (MAT), mean temperature of the warmest quarter (MTW), temperature seasonality (TS), annual precipitation (AP), and precipitation of the wettest quarter (PW; Supplementary Figure 6). Seasonality indicates the variation through the year. Quarter data was included because the populations are situated in a region with Mediterranean climate, which entails rains mainly during winter and dry, warm summers. This seasonal variation results in cacti storing most of their water during winter and showing hydric and temperature stress during summer (Nobel, 1977). More importantly, quarter data can reflect better specific climatic changes over a year than mean climatic variables.

To assess if the parasite negatively affects its host, we compared healthy vs. infected cacti’s functional traits with a MANOVA analysis. The traits used were: fitness, photosynthetic activity (winter and summer separately), stomatal traits (SD, GL, PCI), nutrient (C:N, %C, %N), and isotopic composition (∂^13^C, ∂^15^N). Additionally, linear and generalized mixed models were used to evaluate if fitness and photosynthetic activity were more significantly affected by parasitism, nutrient and isotopic composition, or climate (MAT, MTW, AP, PW), using the populations as the random variable. Because fitness is the count of fruits per cacti, the data was fit with a Poisson distribution in a generalized linear mixed model (GLMM). For the photosynthetic activity, linear mixed models (LMM) were performed separately for winter and summer measurements.

Since *T. aphyllus* is a holoparasite and thus takes all nutrients from the host, we were interested in comparing the host nutrient composition against its parasite. To this end, we contrasted the cactus composition (infected and healthy cacti) against the parasite composition with a MANOVA analysis. Afterward, the separate effect of each parameter was assessed with ANOVAs and additional differences with *a posteriori* Tukey HSD analysis. Further, to determine if the host or climate have an effect over the parasite, GLMM or LMM were performed for each nutrient (%C, %N, C:N) and isotope (∂^13^C, ∂^15^N), with the site as the random factor. For %C and %N, a binomial GLMM was used because percentages are restricted from 0 to 1 (or 0 to 100%). For the rest, LMMs were performed. In the case of C:N ratio and ∂^15^N, there was no effect from the random variable, so a linear model was best fitted. To better visualize any differences, a PCA analysis was conducted between *T. aphyllus*, healthy, and infected *E. chiloensis*. All analyses were done in R (R Core Team, 2018) and models (GLMM, LMM, LM) were calculated with the lmer4 package (Bates et al., 2015). For model selection, we used the “*dredge*” function in the “MuMIn” package (Barton, 2018). Model fit was compared using the bias-corrected Akaike’s Information Criterion (AICc).

## Results

Overall, we found no significant differences between infected and healthy cacti when comparing with a MANOVA test all studied traits [Pillai’s Trace = 0.09, *F*(9) = 1.264, *p* = 0.264]: reproductive fitness, photosynthesis (during winter and summer), stomatal traits (SD, GL, PCI), nutrients (carbon and nitrogen content: C:N, %C, %N), and isotopic composition (∂^13^C and ∂^15^N). However, when we explored the effects of parasitism over the individual traits with linear models, the results obtained were mixed as outlined below.

*Echinopsis chiloensis* stomatal morphology and density showed no differences for parasitism or climate (Supplementary Figure 1). The results were remarkably heterogeneous, with high intra- and inter-population diversity (Supplementary Figures 1D,E) and presenting the classic inverse correlation of stomatal size and density (Supplementary Figure 1C; *R* = −0.33, *p* < 0.001).

**FIGURE 2:**
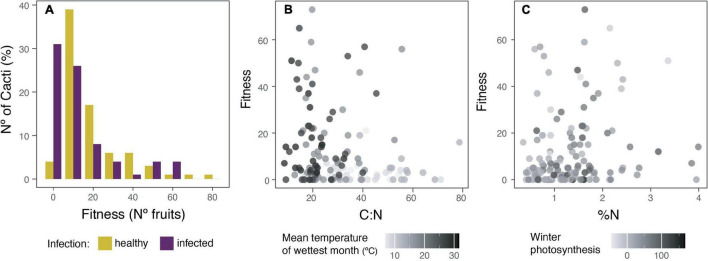
The effect of the holoparasite *Tristerix aphyllus* and climatic conditions over fitness of its host *Echinopsis chiloensis*. Fitness, understood as the number of fruits produced, was better explained in a generalized linear mixed Poisson model (marginal *R*^2^ = 0.28, conditional *R*^2^ = 0.37, AICc = 2102.9) by: infection, the amount of nitrogen on the cortex (%), winter photosynthesis, carbon to nitrogen ratio, and precipitation of the wettest quarter. **(A)** Percentage of healthy (yellow) and infected (violet) cacti producing a specified number of fruits, assuming the same total number of cacti for each. **(B)** Relation of C:N ratio and mean temperature of wettest month with fitness. **(C)** Relation of nitrogen content (%N) and winter photosynthesis with fitness.

For fitness, calculated as the number of fruits produced, a generalized linear mixed Poisson model was used to understand which of the measured variables best explains it. We found that differences in fitness are better explained by: (1) being parasitized, (2) the amount of nitrogen in the cortex (%N), (3) winter photosynthesis, (4) carbon to nitrogen ratio (C:N), and (5) precipitation of the wettest quarter (PW) (marginal *R*^2^ = 0.28, conditional *R*^2^ = 0.37, AICc = 2102.9; Figure 2). Infected plants have 16% fewer fruits than healthy ones, and there was a 42% increase in the number of fruits for each additional increase in N%. Increasing winter photosynthesis, decreasing C:N ratio, and higher PW produced only a ≤1% increment in the number of fruits produced for each additional variable change.

**FIGURE 3:**
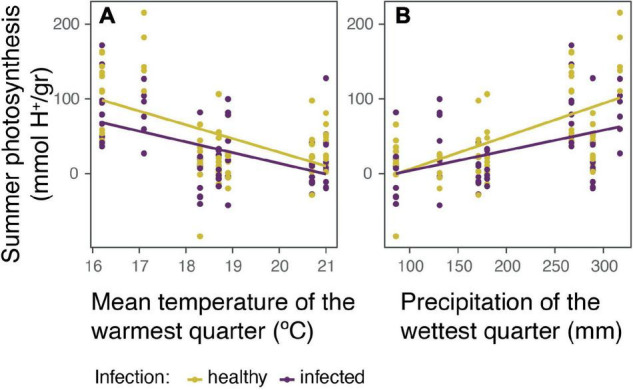
The effect of the holoparasite *Tristerix aphyllus* and climatic conditions over the summer photosynthesis (mmol H+/gr) of its host *Echinopsis chiloensis*. Summer photosynthesis was better explained in a linear mix model (marginal *R*^2^ = 0.5, conditional *R*^2^ = 0.5, AICc = 1255.9) by: infection, **(A)** mean temperature of the warmest quarter (summer), and **(B)** precipitation of the wettest quarter (winter) over cactus photosynthesis. In both cases the effects are separated between healthy and infected cacti.

The photosynthetic rate of healthy and infected cacti was evaluated in the rainy season (winter) and in the dry season (summer). In general, we see a slight negative effect of parasitism over winter and summer photosynthesis (Supplementary Figure 2). However, after analyzing the data with linear mix models, winter photosynthesis did not show significant correlations for any studied variables (Supplementary Table 1). In contrast, summer photosynthesis was better explained by: (1) being parasitized, (2) mean temperature of the warmest quarter (MTW, which is during summer), and (3) precipitation of the wettest quarter (PW, during winter) (marginal *R*^2^ = 0.5, conditional *R*^2^ = 0.5, AICc = 1255.9; Figure 3). Presenting a negative correlation with being parasitized and mean summer temperatures, and a positive correlation with winter precipitations.

**FIGURE 4:**
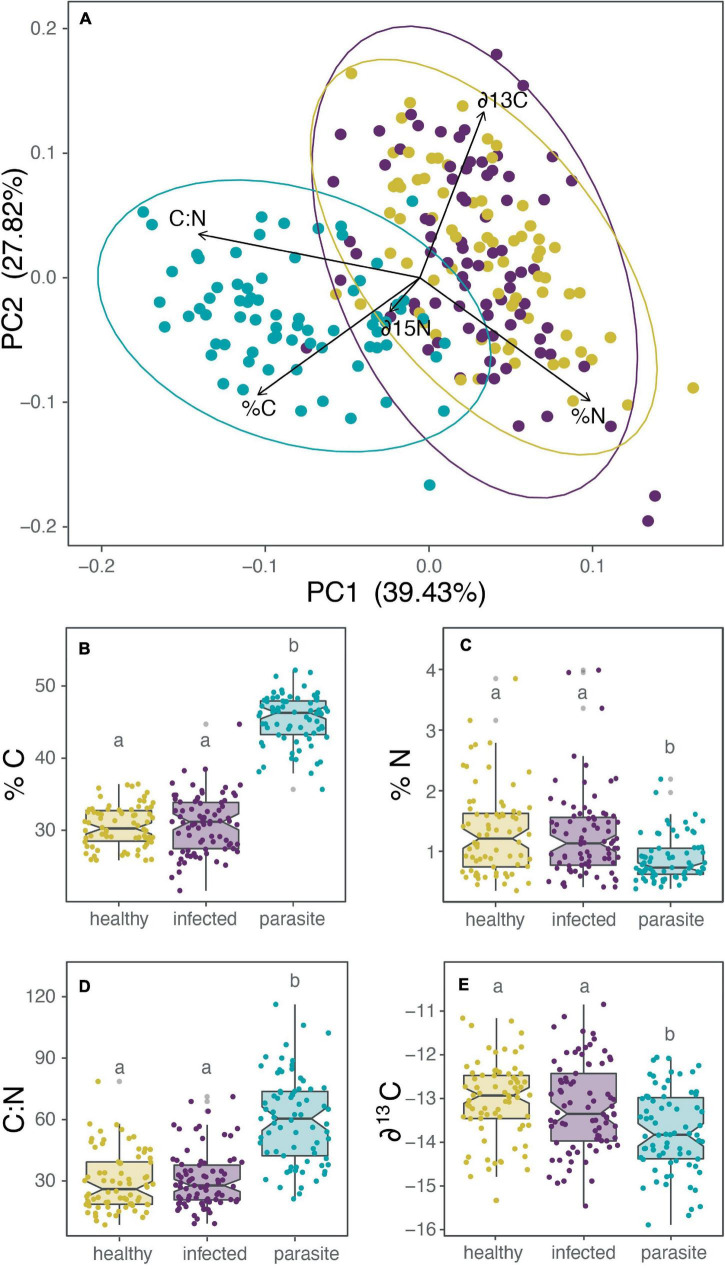
A comparison of the nutrient and isotopic composition between the holoparasite *Tristerix aphyllus* (green) and its host *Echinopsis chiloensis*. In all cases *E. chiloensis* is separated between healthy (yellow) and infected (violet) cacti. **(A)** PCA of nutrients and isotopes, **(B)** boxplot of carbon concentration in the tissue (%C), **(C)** nitrogen concentration in the tissue (%N), **(D)** C:N ratio in the tissue, and **(E)** the isotope ∂^13^C in the tissue. The results for the isotope ∂^15^N are not shown because there were no differences between the parasite, healthy and infected cacti. Separated ANOVAs were performed for each isotopic or nutrient composition variable. Subsequently *a posteriori* Tukey HSD analysis confirmed that all healthy and infected cactus were the same (marked with a), and only the parasite (b) significantly differed (*p* < 0.001 for all cases).

For comparing the isotopic and nutrient composition between parasites, healthy, and infected cacti, we first evaluated the differences with a MANOVA test, from which we obtained significant differences [Pillai’s Trace = 0.88, *F*(444) = 35,064, *p* < 0.001]. Separated ANOVAs were performed for each isotopic or nutrient composition variable (%C, %N, C:N ratio, ∂^13^C, ∂^15^N). Subsequently, *a posteriori* Tukey HSD analysis confirmed that all healthy and infected cactus were the same, and only the parasite significantly differs for the previously mentioned nutrient or isotope (Supplementary Table 2). Additionally, a principal component analysis (PCA) was also employed to visualize the results better (Figure 4A). The PCA’s first and second principal components (PC1 and PC2, respectively) explained 55% of the total variance (Figure 4A), and PC1 can distinctly separate the cactus of the mistletoe despite only explaining 39.4% of the variance. PC1 variance was mainly explained by Carbon content (%C), where the parasite possesses 15% more carbon than the cactus [Figure 4B; ANOVA: *F*(2,225) = 44.61, *p* < 0.001]. Nitrogen content (%N) is significantly different [Figure 4C; ANOVA: *F*(2,225) = 11,755, *p* < 0.001] between the parasite (0.86% N on average) and its host (1.26% N on average). Accordingly, C:N ratio was significantly higher in the parasite [Figure 4D; ANOVA: *F*(2,225) = 67.648, *p* < 0.001]. With respect to the isotopes, the parasite has higher discrimination for lighter carbon isotopes (∂^13^C; Figure 4E and Supplementary Figure 5) from its host pool (−13,162 versus −13,722 on average), but not for nitrogen isotopes (∂^15^N; Supplementary Figure 5).

**FIGURE 5:**
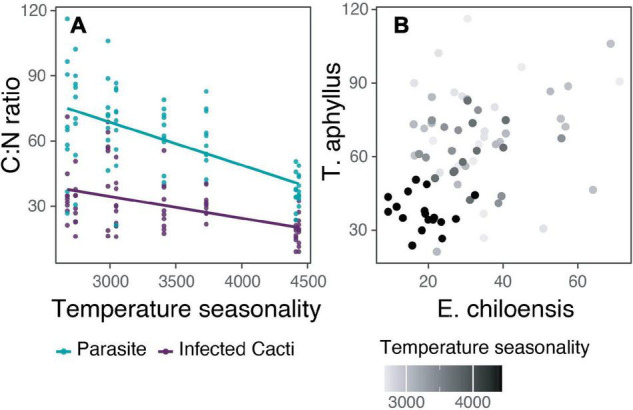
The relation of C:N ratio of the holoparasite *Tristerix aphyllus* with its host *Echinopsis chiloensis* and temperature seasonality. **(A)** Linear relation of temperature seasonality with C:N ratio differentiating between the parasite and infected cacti nutrient content (for the parasite: *R*^2^ = 0.35, *p* < 0.001). **(B)** C:N ratio for each sampled holoparasite compared with its particular host.

Generalized (GLMM) and linear mixed (LMM) models were performed for parasite isotopes and nutrients composition separately (C%, N%, C:N ratio ∂^13^C, ∂^15^N) to examine whether they relate to their host’s isotopic and nutrient composition and the climatic conditions. In cases in which belonging to a population did not explain part of the variance (random factor = population), linear models (LM) were performed. The isotopic composition (∂^13^C, ∂^15^N) and the content of carbon and nitrogen of the parasite were not significantly related to the host nutrient content or with any climatic variable. The only exception was the parasite’s C:N ratio that was best explained by temperature seasonality (TS; LM: *R*^2^ = 0.35, *p* < 0.001). When seasonality is lost, that is, when winter and summer temperatures are more similar (lower TS), the variance of C:N ratio increases, and when winter and summer temperatures differ, the variance of C:N ratio decreases (Figure 5). The same is true for the host’s C:N ratio (LMM: marginal *R*^2^ = 0.23, conditional *R*^2^ = 0.30, AICc = 583.9), even though we did not detect a relationship between the C:N ratio of the host and parasite.

## Discussion

Cacti are highly specialized plants with traits that allow them to inhabit dry areas by optimizing their water use, such as CAM photosynthesis, succulent stems, and sunken stomata while maintaining a water and nutrient reserve in their stem. Given the difficulties of acquiring resources, the infection of the holoparasite *Tristerix aphyllus* seems to have a substantial effect on the fitness of its host *Echinopsis chiloensis*, as was previously reported for other *E. chiloensis* populations (Silva and Martínez del Río, 1996; Medel, 2000). Interestingly, host fitness was not only affected by parasitism. Other factors such as a higher nitrogen content in cactus tissues that increases toward higher latitudes improved the reproductive output of the cacti (Supplementary Figure 4). This increase could be indirectly related to the increase in precipitation to the south. More irrigation could allow higher transpiration rates, with a consequent increase in nitrogen absorption (Fox, 2019; Kunrath et al., 2020). Increased precipitations could also increase nitrogen-fixing bacteria and fungi in more irrigated soils (Amirnia et al., 2019). Unfortunately, we did not measure the nitrogen in soils or the evapotranspiration of cacti. However, the nitrogen levels are incredibly high in the soil around our studied areas, exceeding 80 mg/Hg (Nendel et al., 2019). Additionally, Mizrahi et al. (2007) reported that more irrigated cacti produced more fruits, so the increase in fruit production in southern populations could be due to the same phenomena. Thus, the increased nutrient ratio and water availability could allow the host to invest more resources in reproduction, independently of parasitism. These results suggest that if the current drought suffered by the Chilean Mediterranean area continues, it could strongly restrict the fitness of *E. chiloensis* in the future (Araya-Osses et al., 2020). Still, more studies are needed to untangle the nitrogen and precipitation effect and determine if there is also latitudinal nitrogen or microbiota soil gradient.

Regarding photosynthesis, *E. chiloensis* decreased its photosynthetic rate with parasitism, but these differences were significant only during the dry season (summer). Scalon et al. (2021) also reported a reduction in the host photosynthesis due to the hemiparasite of the Loranthaceae family *Passovia ovata*, although only during the wet season. The seasonal differences could result from the distinct nature of the parasites (holoparasite vs. hemiparasite), together with the ability of cacti to store sugars in their stem which increases during the wet season. Other holoparasites such as *Cuscuta gronovii*, *C. campestris*, or *C. australis* have also recorded a significant reduction in the photosynthesis of their host under dry conditions (Shen et al., 2007; Evans and Borowicz, 2013, 2015; Le et al., 2015). Interestingly, the contrast between healthy and infected cacti increased in higher irrigated populations (Figure 3), which are the ones under a weaker precipitation seasonality. Even though overall photosynthesis increases when there is more water available, the parasite seems to restrict the rate of increase. Yet, there is no clear pattern when winter and summer photosynthesis are compared along the latitudinal gradient (Supplementary Figure 2). These results add to a growing body of evidence on the strong effect of precipitation seasonality on CO_2_ assimilation in cacti, although the direction in which they relate is dependent mainly on the climate region (Pimienta-Barrios et al., 2000, 2002; Ricalde et al., 2010; Zañudo-Hernández et al., 2010; Bronson et al., 2011).

Even though *T. aphyllus* draws all its water and nutrients from cacti for survival, the nutrients concentration between parasite and host was considerably different (Figure 4). However, the cacti did not show differences in nutrient composition or isotopes between healthy and infected individuals. Carbon content (%C) showed the broadest differences among the nutrients measured, present in greater amounts in the parasite than in its host. There are two potential reasons behind these differences: firstly, the distinctive nature of the tissue sampled in the parasite (reproductive: flower pedicels) and in the host (photosynthetic: cortex), and secondly, because the parasite may be acting as a sink (Jeschke and Hilpert, 1997; Hibberd et al., 1998; Press et al., 1999; Birschwilks et al., 2006). When considering that *T. aphyllus* is entirely dependent on host carbon stock for growing and respiration, explaining the lack of differences in carbon concentrations between healthy and infected cacti can be challenging. Even more when no overcompensation in photosynthesis is observed to counteract carbon depletion by the parasite; on the contrary, parasitism tends to decrease the photosynthetic rate. However, since cacti act as a nutrient storehouse, parasite may be using recently acquired and stored nutrients, without causing differences in carbon content between healthy and infected cacti. To perceive a considerable impact on the cacti’s physiology would require a more substantial effect of the parasite, which is more likely to happen with greater parasite loads.

Contrary to what was observed with carbon concentrations, nitrogen content was higher in the host than the parasite, with differences slightly accentuated in southern populations (Supplementary Figure 4). The presence of a photosynthetic apparatus in the host that is missing in the parasite might be one reason for this difference. Still, our results coincided with reports of different hemiparasite mistletoes in New Zealand, where the concentration of nitrogen in the parasite tissue decreases as it increases in the host’s tissue (Bannister and Strong, 2001). The trend reverses when the nitrogen concentration in the host tissue is limited (Bannister and Strong, 2001), which is not the case for *E. chiloensis*.

As expected, carbon (%C) and nitrogen (%N) content influence the C:N ratio in the tissue, being significantly higher in the mistletoe because of higher %C and lower %N than in its host. These could reflect a closer connection to the phloem than to the xylem, which was partially confirmed by Mauseth et al. (1984). They reported that *T. aphyllus* filaments are more abundant in the phloem vascular tissues because it continuously invades it, whereas the xylem is invaded episodically. This also reflects the high dependency of the mistletoe on carbon for growth and a lower dependence on nitrogen, as it lacks photosynthetic tissues. On the other hand, the C:N ratio increases its variance when the temperatures seasonality decreases, regardless of latitude.

For the isotopic signature, we expected to obtain enriched or depleted values for the parasite compared to the host but always maintaining a correlation between both across populations. Accordingly, ∂^15^N showed a similar although not significant trend between parasite and host in all populations studied (Supplementary Figure 2), supporting the idea that mistletoe nitrogen is completely acquired from its host (Schulze et al., 1984; Bannister and Strong, 2001). The same results were reported for holoparasitic species of the genus *Hydnora* (Bolin et al., 2010) and the hemiparasite *Tapinanthus oleifolius* when they parasitize different hosts species (Schulze et al., 1991). CAM holoparasitic plants have been reported to be slightly more negative in ∂^13^C values than their host (between 0.55 and 1.5‰, see De la Harpe et al., 1981; Ziegler, 1994; Cernusak et al., 2004; Bolin et al., 2010). *T. aphyllus* follows this trend and presents values of enriched mistletoe tissues ranging between 0.63 and 1.42‰ (Figure 4B). Ziegler (1996) had already reported enriched ∂^13^C values for mistletoes in our northernmost population (Tololo, Supplementary Figure 5: TO). Interestingly, we reported an increase of 6% for *T. aphyllus* and 9% for infected *E. chiloensis* for the Tololo locality when compared to Ziegler’s results, which could be explained by the increase in atmospheric carbon accumulated in the last 20 years.

Taken together, our results showed that the infection by *T. aphyllus* does not have a profound effect on the physiology of its host *E. chiloensis*, despite causing a decrease in its reproductive fitness and photosynthetic rate. Regardless of parasitism, the cacti’s fitness and physiology were favored by wet winters, dry summers, and places exhibiting marked seasonality. Consequently, we confirm the environmental dependence of the effect of the parasite on its host, opening new and exciting research questions.

## Data Availability Statement

The original contributions presented in the study are included in the article/Supplementary Material, further inquiries can be directed to the corresponding authors.

## Author Contributions

CO and FP conceived the ideas and design methodology. CO collected the data with the assistance from DA-M. DA-M and MM analyzed the data. CO and DA-M led the writing of the manuscript. All authors contributed critically to the drafts and gave final approval for publication.

## Conflict of Interest

The authors declare that the research was conducted in the absence of any commercial or financial relationships that could be construed as a potential conflict of interest.

## Publisher’s Note

All claims expressed in this article are solely those of the authors and do not necessarily represent those of their affiliated organizations, or those of the publisher, the editors and the reviewers. Any product that may be evaluated in this article, or claim that may be made by its manufacturer, is not guaranteed or endorsed by the publisher.
