# Supplementary material for: The Physiological Effect of a Holoparasite Over a Cactus Along an Environmental Gradient
Source: Front Plant Sci. 2021 Nov 18;12:763446. doi: 10.3389/fpls.2021.763446 (PMC8636672; doi:10.3389/fpls.2021.763446)
Supplement: Supplementary file 1 [file Data_Sheet_1.docx]

**Supplementary material**

Table S1. Results of linear mixed models for winter photosynthesis of *Echinopsis chiloensis* while being parasitized by *Tristerix aphyllus*. Values are derived from multiple mixed models using a set of different explanatory variables based on the infection (healthy or infected cacti), the isotope ∂15N in the cactus tissue, nitrogen content in the cactus tissue (%N), and two macroclimatic variables (mean annual temperature MAT, and annual precipitation AP), while using the population as a random factor. “NA” values are due to the absence of the respective fixed effect in the best model (∆AICc<2). The best fitted model (lowest AICc) is the null model (with no variables). The R^2^ are marginal (R^2^m) that represents the variance explained by the fixed effects, and conditional (R^2^c) that is interpreted as a variance explained by the entire model, including both fixed and random effects.

| Intercept | Infection | ∂15N | %N | MAT | AP | df | logLik | AICc | weight | R2m | R2c |
| --- | --- | --- | --- | --- | --- | --- | --- | --- | --- | --- | --- |
| 42.99 | NA | NA | NA | NA | NA | 3 | -691.7 | **1389.6** | 0.38 | **0.00** | 0.18 |
| 47.41 | -8.26 | NA | NA | NA | NA | 4 | -690.8 | **1389.9** | 0.32 | **0.01** | 0.20 |
| 39.45 | NA | NA | 2.71 | NA | NA | 4 | -691.6 | **1391.4** | 0.15 | **0.00** | 0.17 |
| 65.47 | NA | NA | NA | -0.16 | NA | 4 | -691.6 | **1391.5** | 0.14 | **0.01** | 0.18 |

Table S2. Results from Tukey HSD comparing nutrients and isotopic composition on the tissues of *E. chiloensis* being healthy and infected, and its holoparasite *T. aphyllus*. The variables where: carbon content tissue (%C), nitrogen content (%N), isotopic composition of ∂13C, and ∂15N. In bold the significant differences between each pair.

| Variable | Comparison | Mean difference | Lower | Upper | p |
| --- | --- | --- | --- | --- | --- |
| %C | ***Parasite-Infected*** | 14.5276666 | 13.207928 | 15.847405 | **0.0000000*** |
|  | *Healthy-Infected* | -0.2971113 | -1.612151 | 1.017928 | 0.8551715 |
|  | ***Healthy-Parasite*** | -14.8247779 | -16.173780 | -13.475776 | **0.0000000*** |
| %N | ***Parasite-Infected*** | -0.38913745 | -0.6257156 | -0.1525593 | **0.0004010*** |
|  | *Healthy-Infected* | 0.05447603 | -0.1812599 | 0.2902119 | 0.8490258 |
|  | ***Healthy-Parasite*** | 0.44361348 | 0.2017894 | 0.6854376 | **0.0000670*** |
| C:N | ***Parasite-Infected*** | 29.5756912 | 23.166602 | 35.98478 | **0.0000000*** |
|  | *Healthy-Infected* | -0.6968616 | -7.083133 | 5.68941 | 0.9641288 |
|  | ***Healthy-Parasite*** | -30.2725528 | -36.823759 | -23.72135 | **0.0000000*** |
| ∂13C | ***Parasite-Infected*** | -0.5613952 | -0.9227320 | -0.2000585 | **0.0008956*** |
|  | *Healthy-Infected* | 0.1433734 | -0.2166769 | 0.5034237 | 0.6158928 |
|  | ***Healthy-Parasite*** | 0.7047686 | 0.3354195 | 1.0741177 | **0.0000321*** |
| ∂15N | *Parasite-Infected* | 0.36853036 | -0.606576 | 1.3436367 | 0.6461150 |
|  | *Healthy-Infected* | -0.06919086 | -1.040826 | 0.9024439 | 0.9845600 |
|  | *Healthy-Parasite* | -0.43772121 | -1.434450 | 0.5590075 | 0.5548819 |


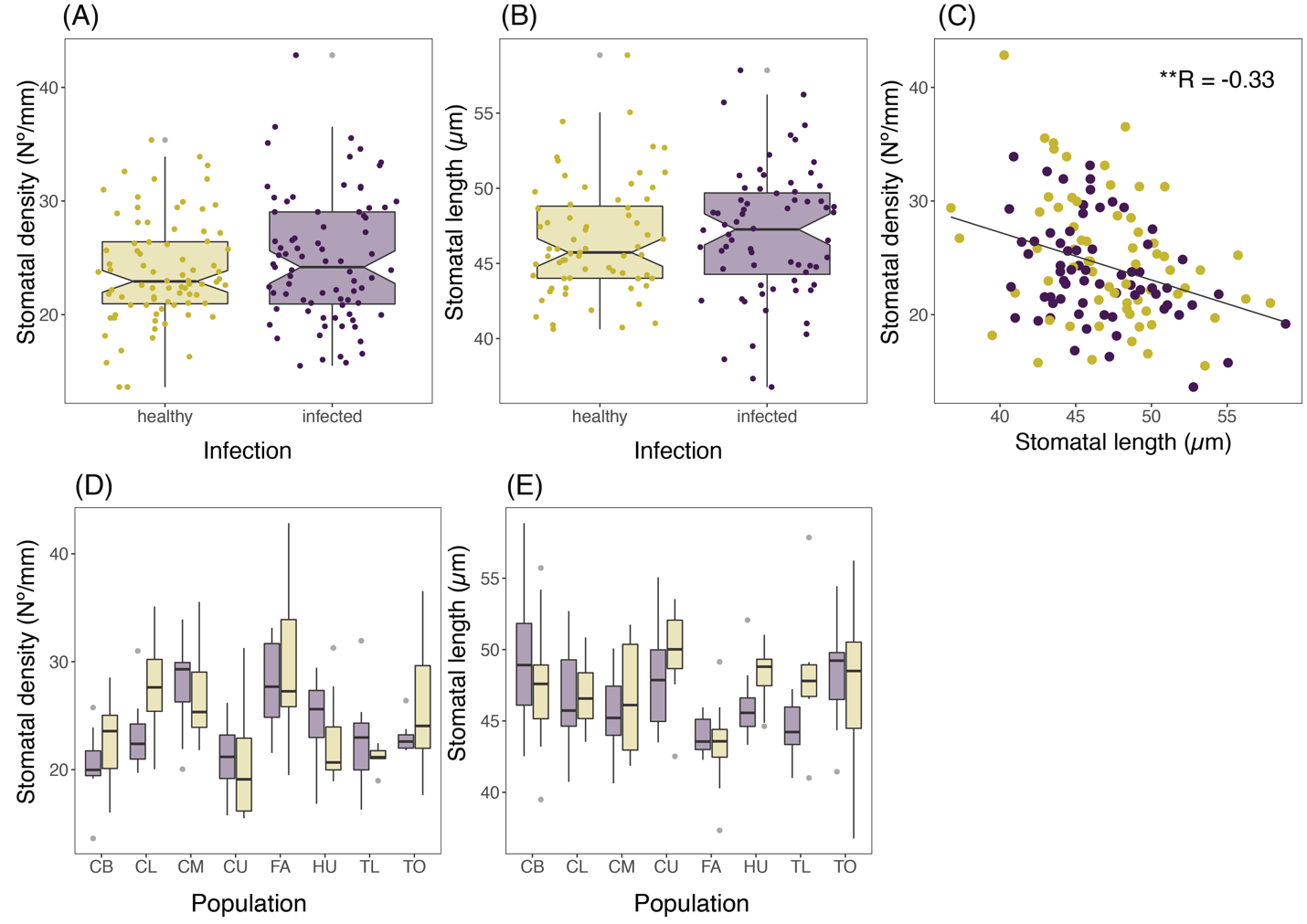


Figure S1. Comparison of stomatal traits between healthy (yellow) and infected *E. chiloensis* (violet) by the holoparasite *T. aphyllus*. (A & D) Stomatal density (Nº/mm^2^), (B & E) stomatal length (µm), (C) stomatal length to stomatal density of each individual. The populations are ordered by latitude from north to south.


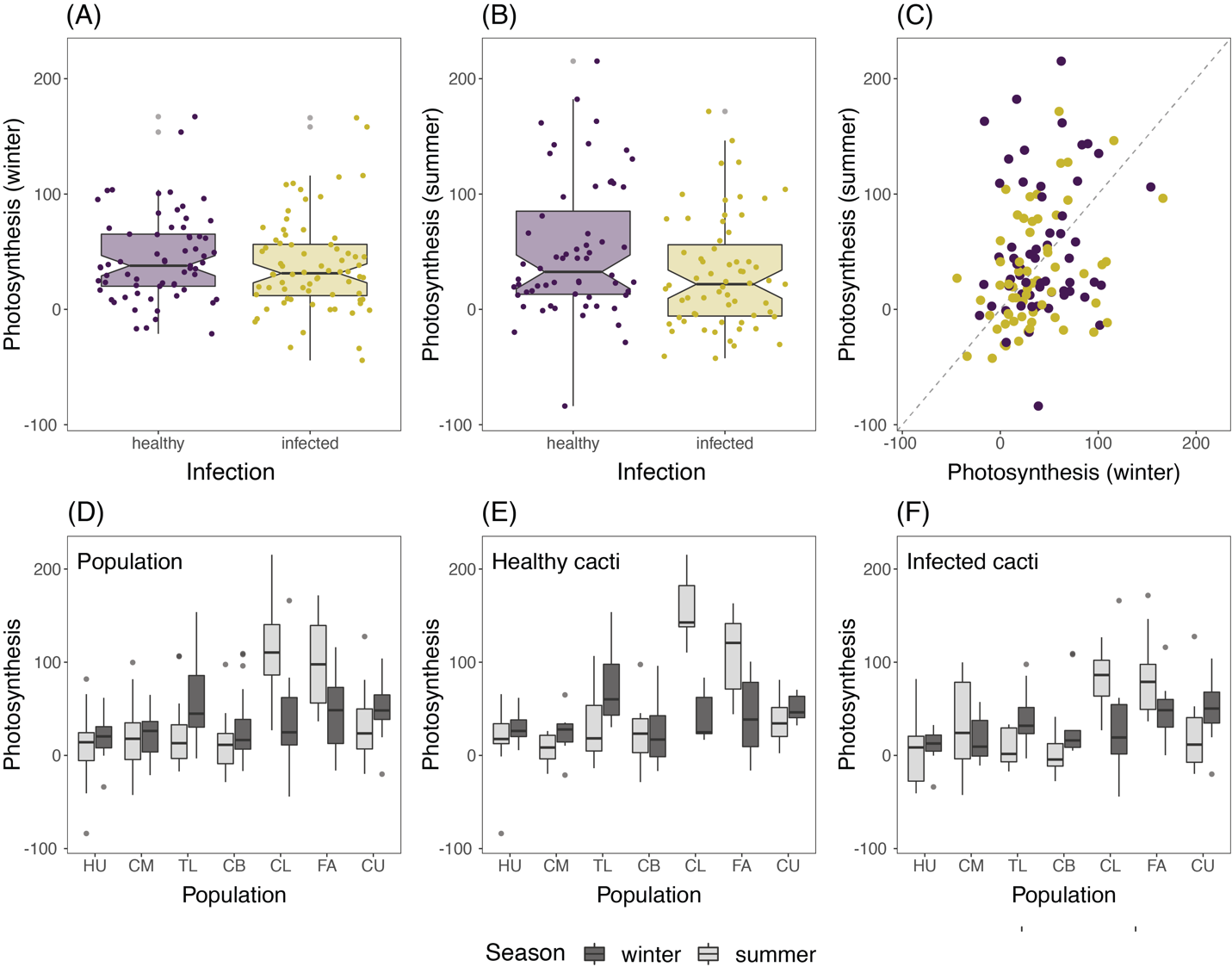


Figure S2. Comparison of photosynthesis (mmol H+/gr) between healthy (yellow) and infected *E. chiloensis* (violet) by the holoparasite *T. aphyllus*. (A) Winter photosynthesis, (B) Summer photosynthesis, (C) Summer to winter photosynthesis of each individual, (D) winter and summer photosynthesis of *Echinopsis chiloensis* in an environmental gradient given by latitude, which include both healthy and infected cacti by the holoparasite *Tristerix aphyllus*, (E) only healthy cacti, and (F) only infected cacti. The populations are ordered by latitude from north to south.


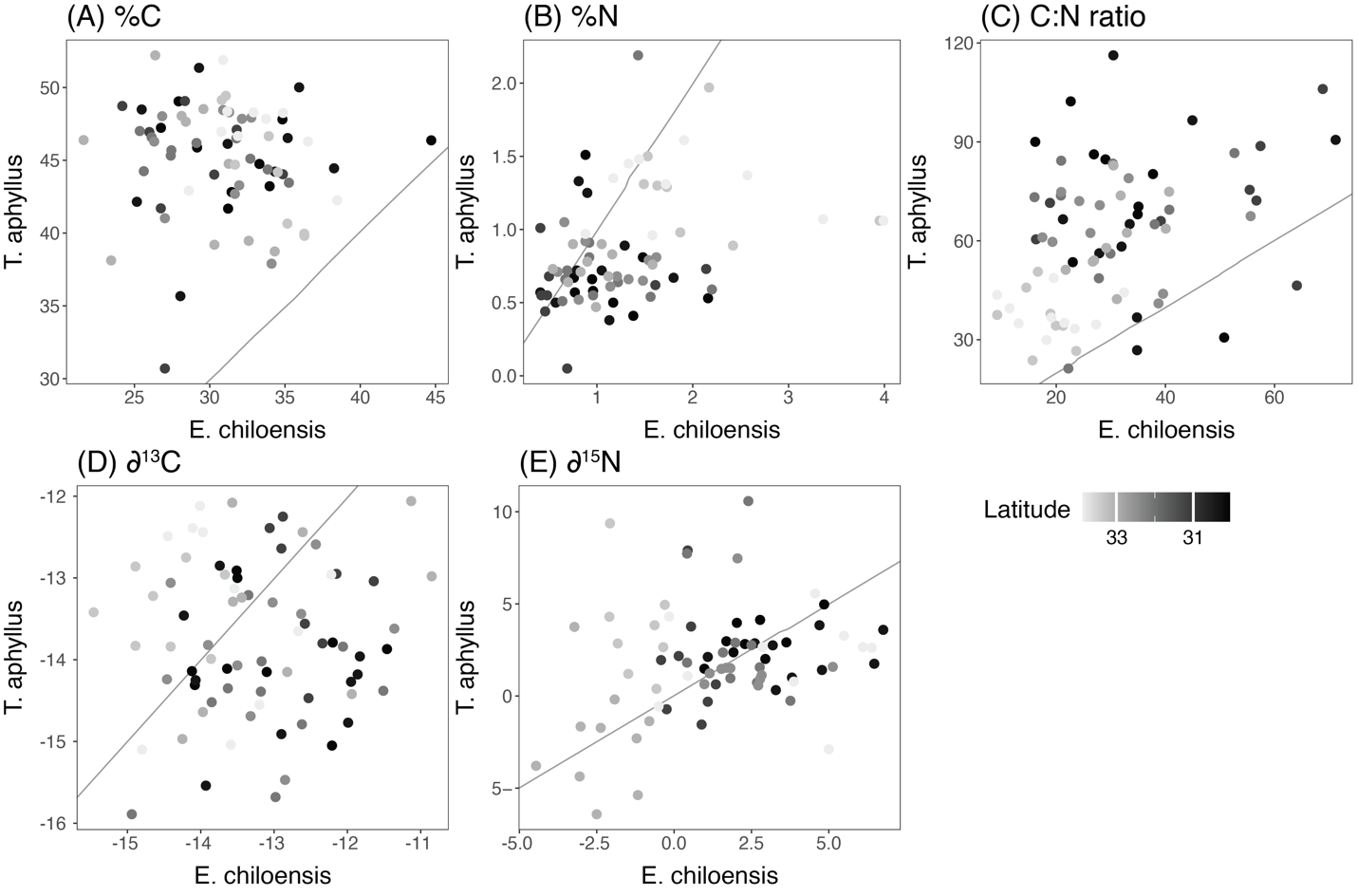


Figure S3. Isotope and nutrient composition of the parasite *T. aphyllus* versus its host *E. chiloensis* throughout an environmental gradient given by latitude. (a) carbon content, (b) nitrogen content, (c) C:N ratio, (d) ∂13C content and, (e) ∂15N content. The greyscale represents the geographical latitude distribution of the population each individual belongs to. The line is signaling the 1:1 concentration.


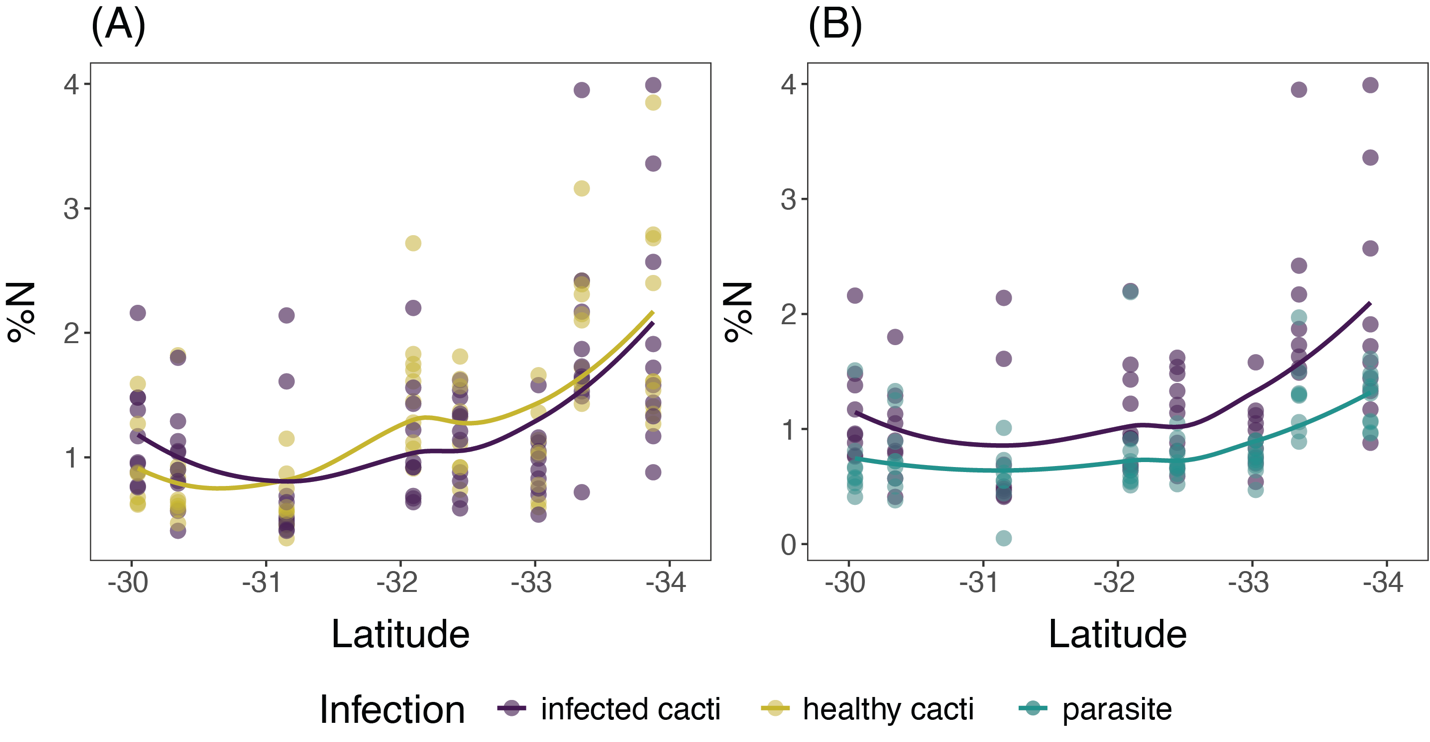


Figure S4. Concentration of nitrogen in the tissue of the holoparasite *Tristerix aphyllus* and its host *Echinopsis chiloensis* against latitude. (a) Healthy versus infected cacti, and (b) infected cacti versus the parasite. The line is a “loess” approximation of the mean of each population.


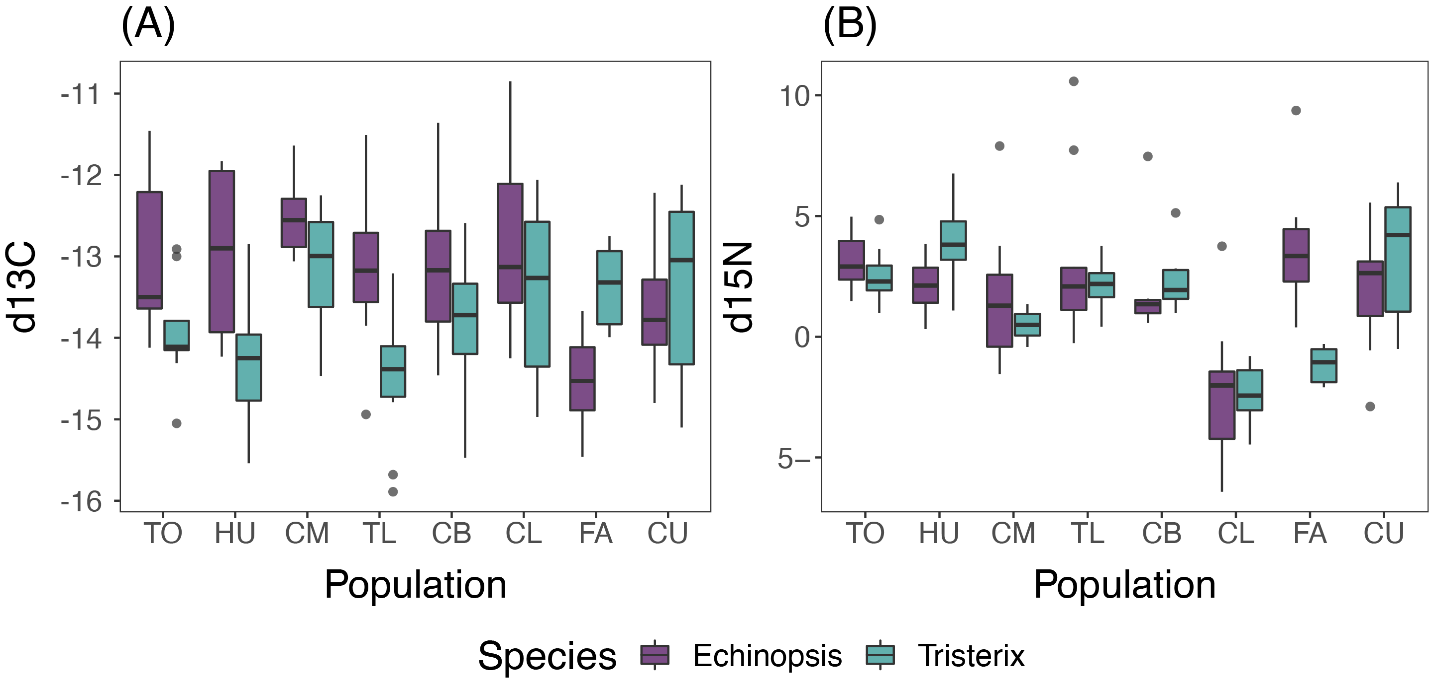


Figure S5. Isotope ∂13C and ∂15N in the tissue of the holoparasite *Tristerix aphyllys* and its host *Echinopsis chiloensis*, per studied population ordered from north to south. This populations are representing an environmental gradient given by latitude.


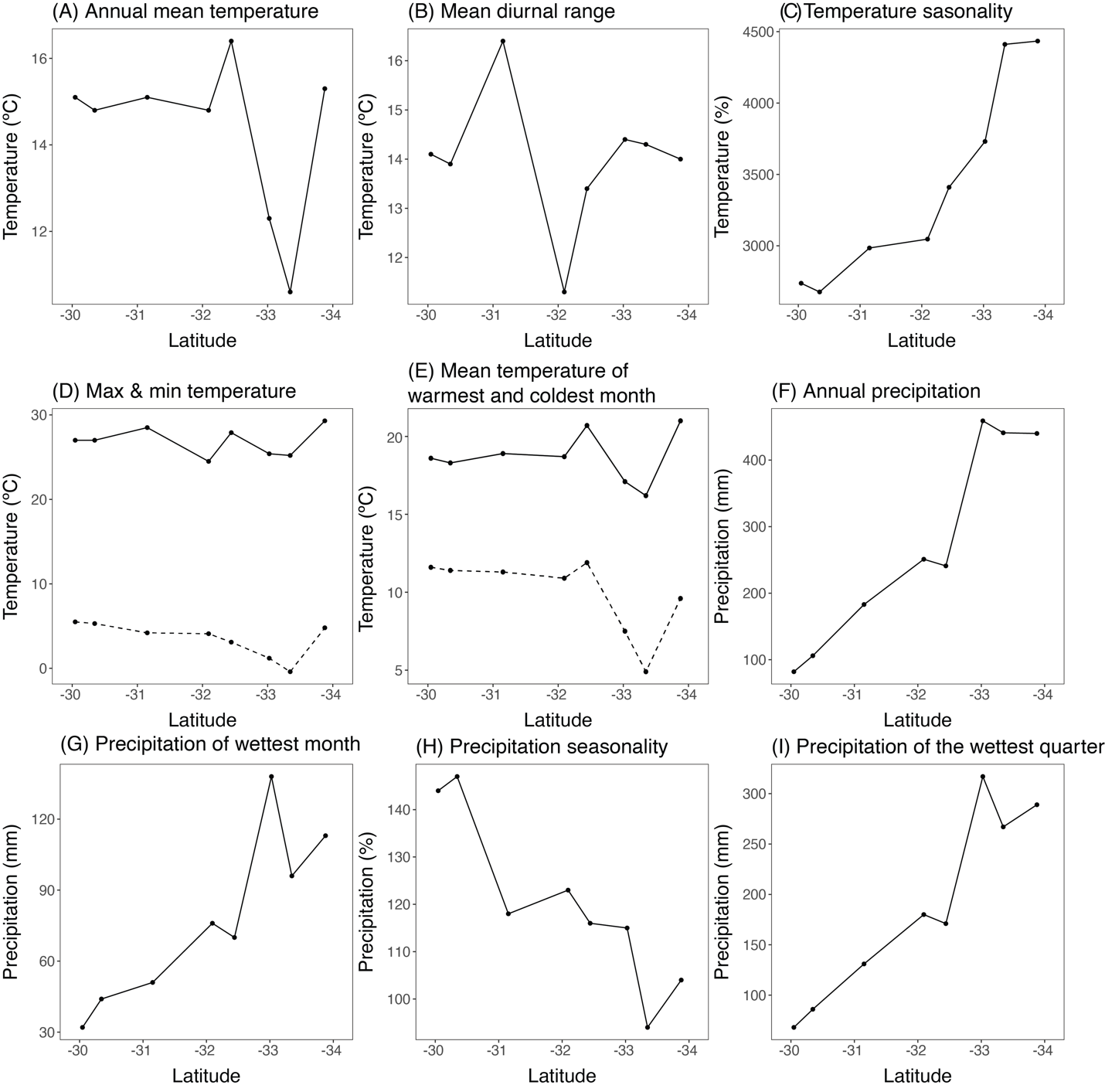


Figure S6. Climatic variables for each studied population of a parasite-host interaction ordered by latitude. The dots represent the populations.
